# Theories and Explanations in Psychology

**DOI:** 10.3389/fpsyg.2019.00958

**Published:** 2019-04-30

**Authors:** Anna M. Borghi, Chiara Fini

**Affiliations:** ^1^Department of Dynamic and Clinical Pyshcology, Sapienza University of Rome, Rome, Italy; ^2^Institute of Cognitive Sciences and Technologies, Italian National Research Council, Rome, Italy

**Keywords:** explanation, computational modeling, embodied and grounded cognition, cross-cultural, theory

Research in psychology has undergone many changes in the last 20 years. The increased and tighter relationship between psychology, neuroscience, and philosophy, the emergence and affirmation of embodied and grounded cognition approaches, the grow of interest on new research topics, the strengthening of new areas, such as the social, cognitive, and affective neuroscience, the spread of Bayesian models, and the recent debates on the replication crisis, represent some of the pieces of the new emerging landscape.

In spite of these novelties, one character of the discipline remains stable: its focus on empirical investigation. While we think this is an important and distinctive feature of our discipline, all too often this fascination for empirical data is accompanied by the absence of an equally deep interest for theory development. Noteworthy, while other scientific disciplines are endowed with a theoretical branch—think of the role of “theoretical physics” for physics—psychology does not have an equally institutionalized theoretical branch. This lack of theoretical interest is testified also by the fact that only few journals (Frontiers represents an exception) accept theoretical articles, i.e., articles that systematize existing evidence to inform a model or develop a new theory.

Here we argue that a strongly theoretical approach, that takes into account and seeks to identify the mechanisms underlying brain and mental processes, and that aims to build formal theories and computational models, can contribute to address the current limitations of psychological research helping it to face important challenges.

In the following we will highlight some limitations of psychological research, that a strong theoretical and philosophically informed approach can contribute to face.

1. **Interdisciplinary dialogue**. True interdisciplinary research is crucial for our understanding of the mind and brain mechanisms. Initially cognitive science was a highly interdisciplinary project, but part of this original richness has gone lost. Yet, complex phenomena such as mental and cognitive processes can be understood only starting from multiple perspectives. However, integrating these perspectives is all but easy. It is therefore crucial to promote solid and reliable interdisciplinary research. This might be done through the creation of novel interdisciplinary structures/department, or through funding policies that privilege research and projects with interdisciplinary teams (the European Community has done some attempts in this direction). In general, we should aim at increasing and promoting the occasions where researchers from different areas debate, developing a common language. Aside from conferences and workshops, journals with interdisciplinary character and interdisciplinary debates represent a fundamental mean to boost this kind of approach.
  A pivotal role in promoting interdisciplinarity can be played by philosophy—and in particular by philosophy of mind and language, philosophy of psychology, philosophy of neuroscience, and of cognitive science. Although not a natural science, empirically-informed philosophy can play a crucial integrative role, helping to build a more comprehensive view of the field and identifying links that cross disciplinary boundaries.
  In a different way, computational models and simulations are instruments that boost interdisciplinarity: a good model has a cumulative character, it helps in theory building and in validation of empirical data coming from different sources and disciplines and obtained with different methods (Caligiore et al., 2010; Pezzulo et al., 2011).
2. **Emphasis on theoretical aspects**. One of the limitations of psychology, that the relationship with philosophy can help reducing, is the scarce emphasis on theories. Being good scientists in psychology and neuroscience increasingly equates with having good methodological competences, and being able to use many techniques and instruments. While we clearly do not intend to under-evaluate the importance of technical mastery and of methodological rigor and competence, we fear that stressing only these aspects can lead young researchers to focus more on very specific topics and paradigms, thus losing the big picture. Instead, we are convinced that research should be guided by a strong theoretical background: solid theories open new perspectives and research questions and might lead to clear and testable research questions. Prioritizing empirical results over theory can instead lead to a growing tendency to obtain fragmented and conflicting evidence about a phenomenon. Psychology (or at least most of its branches), should mainly remain an experimental discipline. However, scientists who do not possess a comprehensive view and focus only on experimental evidence can be very productive but might not lead to the identification of core principles, useful for a real progress in research. Curiously one of the papers that has more deeply influenced and inspired research in embodied cognition, the BBS paper by Barsalou (1999), was not directly founded on empirical evidence—although it discussed empirical evidence related to some of its claims.
  One of the most debated issues in recent psychological research is the replication crisis, and in particular the reliability of scientific results. We do not intend to under-evaluate the attempts to strengthen the results and we really appreciate the recent tendency to distinguish confirmatory and exploratory research. We namely think that research in psychology needs to follow two strategies: a more explorative, and a more confirmative one-science has indeed two different sides, a creative and a monitoring one. Exerting either an inductive or a deductive logic might allow to build strong and reliable empirical-based psychological models.
  However, we think that more resources and more effort should be devoted to find explanations of phenomena [see Van Rooij (2018) for convincing arguments on this]. As argued by Cummins (2000), “a substantial proportion of research effort in experimental psychology isn't expended directly in the explanation business; it is expended in the business of discovering and confirming effects.”
  Based on these considerations, we believe that more emphasis should be given to the ability to plan and construct experiments with the aim of theory building rather than simply to find reliable effects. Not only the tendency to publish unsound results, but also the tendency to search “originality” at all costs without theory should be contrasted. This approach should clearly impact training, education, and also selection of young researchers.
  The objective of ascribing a more crucial role to the theoretical foundations of our discipline can be reached in multiple ways. We will mention just a couple of them.
  One possible strategy is to focus more on core mechanisms, adopting a synthetic approach. As clearly explained by Hommel and Colzato (2017) in a recent grand challenge, a mature science should “learn to value theoretical frameworks that track down core mechanisms in as many phenomena as possible,” and a more parsimonious approach should be promoted. One example is the role played by goals in influencing action representation, imitation, etc.
  Another way to promote a synthetic approach consists in fostering the use of computational models. Computational models can namely help formulating clearer experimental hypotheses, refining theories, and validating them. Particularly promising are dynamic systems approach, neural networks models, Bayesian models.
3. **Epistemological awareness**. It is important to understand where the field is going. Recent years have seen a variety of pivotal changes and modifications. The spread of embodied and grounded cognition has represented a real revolution in the areas of cognition and social cognition, and has determined an important paradigm change. In addition, we have assisted to the introduction of extended mind proposals, the increased role of social neuroscience and overall the development of a very tight relationship between psychology and neuroscience, the increased importance of Bayesian models. Experiments in psychology typically support or disconfirm field theories, but in many cases they do not explicitly relate to these more general approaches or theories. We instead believe that it is important for scientists to situate their own research within a broad theoretical framework, a general theory; this can namely help to form a cumulative baggage of knowledge.
4. **Key methodological challenges of psychology**. The hotly debated replication crisis in science has been particularly deep in psychology. Addressing it in a proper way certainly requires methodological improvements, but also a clear epistemological vision of the specificity of psychological research. The field is divided between scientists who think it is important to address them improving replication, and scientists who think that research should be more focused on innovation and discovery. A strong theoretical approach can provide means to address this crisis, adopting synthetic methods that facilitate the identification of some basic mechanisms rather than focusing on a variety of more or less fashionable effects. It is important to foster debate on this topic, since its outcome may influence the future of our discipline. In addition, it is important to promote the discussion and use of different kinds of computational models, aimed at strengthening theoretical approaches.
5. **Cross-cultural research**. Scholars are starting to recognize that psychological processes are far from being universal (Henrich et al., 2010; Prinz, 2012; Barrett, 2017; Hruschka et al., 2018). As a consequence, psychologists are starting to propose more and more cross-cultural research. The research instruments we now possess, allowing to perform online experiment, allow scientists to perform more easily studies that include multicultural samples. It is important to promote these practices, encouraging researchers of different nations and backgrounds to collaborate. Old debates, such as that related to the influence of language and languages on cognition, have acquired a new fresh status. A mature reflection on these topics is important and crucial for the development of our discipline. Cross-cultural research should be promoted and fostered.
6. **Dear old themes**. Some topics are crucial for the understanding of mind, brain, behavior. Unluckily debates on some apparently old-fashioned topics are sometimes abandoned, but focusing on them can offer new insights and open new research venues. A strong theoretical and philosophical approach, firmly based on scientific evidence, can offer fresh perspectives and exciting new ideas on these topics. Some examples include the nature-nurture debate; the role of notions such as simulation and representation for psychological understanding; the mechanisms underlying the ability of abstraction/abstractness in animal and human cognition; how concepts are acquired and represented in the brain; the effects of language on perception, categorization, and thought; our sense of body; the theories of narrative self; the role of interoceptive and emotional experience, of religious experience, of mindfulness; penetrability of cognition/perception; how consciousness work; the mechanisms underlying the formation of beliefs and their influence on decision making; how we represent others, e.g., through stereotypes and implicit biases, comprehend them, e.g., through mindreading and perspective taking, and act with them, e.g., through joint action; how we represent morality, social norms, and institutions.

To conclude: How do we dream the psychology of the future? First, we dream of a psychology focusing on theoretically solid, explanation-based accounts, and on the identification of key principles rather than on fashionable effects. Second, we dream of a psychology open to diversity,-characterized by an interdisciplinary approach, and more open to methodological contaminations and to the possibility to perform studies on different populations.

## Author Contributions

Both authors listed have made a substantial, direct and intellectual contribution to the work, and approved it for publication.

### Conflict of Interest Statement

The authors declare that the research was conducted in the absence of any commercial or financial relationships that could be construed as a potential conflict of interest.
